# Supplementary material for: Monascuspiloin from Monascus-Fermented Red Mold Rice Alleviates Alcoholic Liver Injury and Modulates Intestinal Microbiota
Source: Foods. 2022 Sep 30;11(19):3048. doi: 10.3390/foods11193048 (PMC9564352; doi:10.3390/foods11193048)
Supplement: Supplementary file 1 [file foods-11-03048-s001.zip › foods-1846983-supplementary.pdf]

Supplementary Material

1 Supplementary Experimental Method

The analysis method of *Monascus* azaphilone pigments

The analysis was conducted by Agilent 2000 HPLC system (Agilent Technologies, Santa Clara, CA, USA) comprised of a binary pump, an autosampler, and a thermostatically controlled column compartment. Pigments were separated on a 250 × 4.6 mm ODS18 column (5μm, Waters, Milford, MA, USA) with a linear gradient of the mobile phase of solvent A (water, containing 0.1% formic acid, v/v) and solvent B (acetonitrile), a flow rate of 1.0 mL/min, oven temperature of 35℃, run time for 55 min. Mass spectrometry was operated with a 6520 QTOF-MS (Agilent Technologies, Santa Clara, CA, USA) equipped with an ESI source. The parameters of the ESI source were set as follows: capillary voltage, 3500 V; fragmentor voltage, 135 V; drying gas (N_2) temperature, 350 °C; drying gas (N_2) flow rate, 10.0 L/min; nebulizer gas pressure, 30 psig; OCT RF V, 750 V; skimmer voltage, 65 V. Each sample was analyzed in positive modes with a mass scan range of m/z 50–1200 Da, and collision energy (CE) were set as 15 and 30 eV. Data acquisition and analysis were operated by Agilent LC/MS Qualitative Analysis Software (version B.04.00).

2 Supplementary Figures

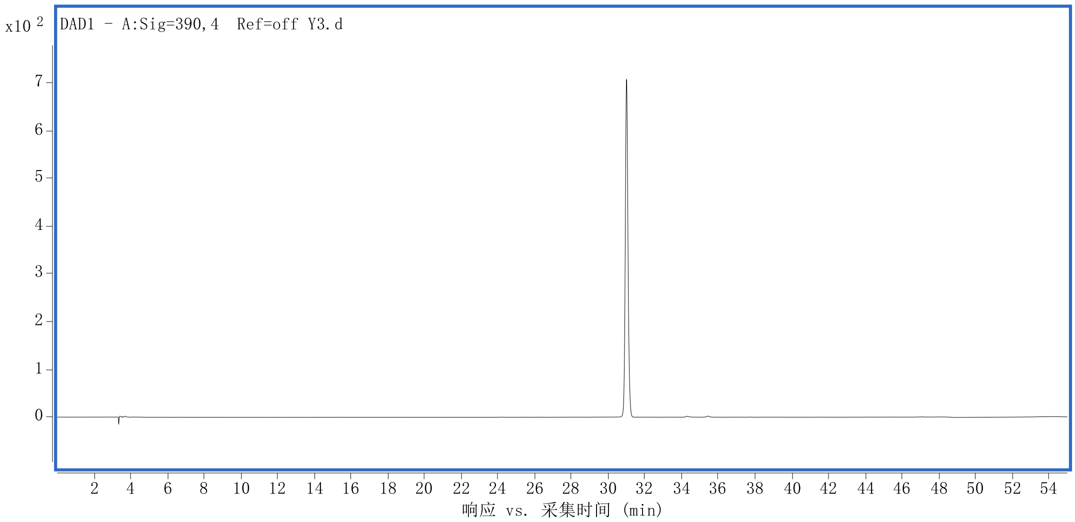

Figure S1. High performance liquid chromatogram of monascuspiloin at 390 nm.

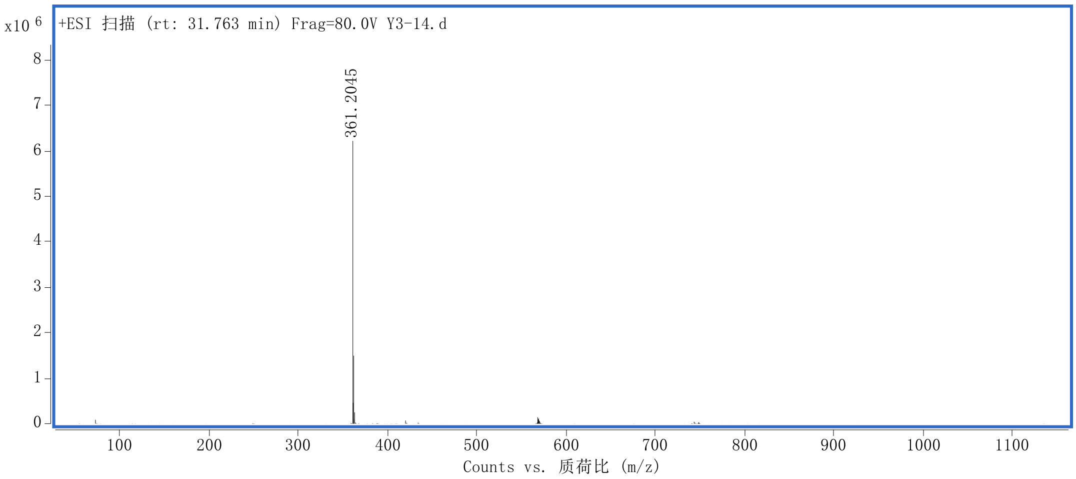

Figure S2. Primary mass spectrum of monascuspiloin (molecular weight: 360.20)

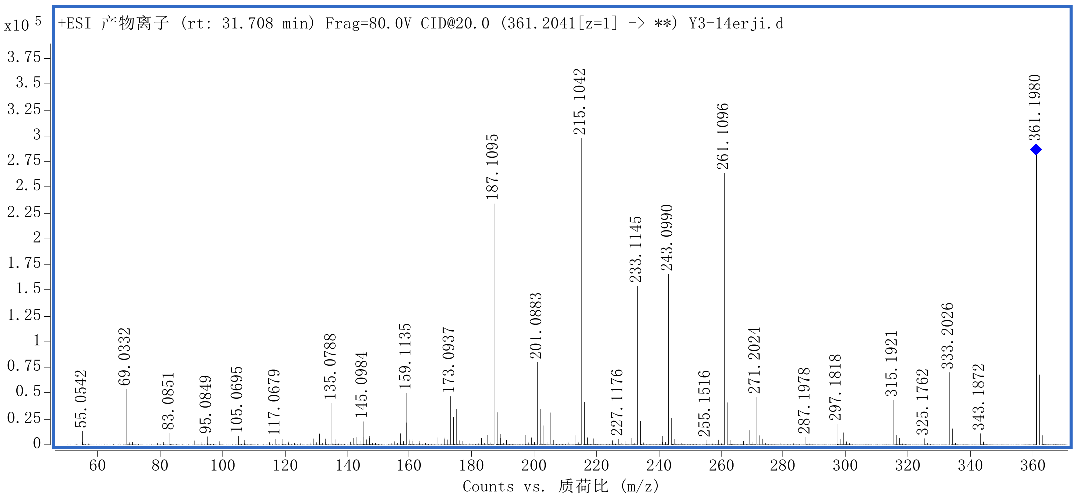

Figure S3. Secondary mass spectrum of monascuspiloin (MS2: 315.1947; 271.1963; 243.1012).

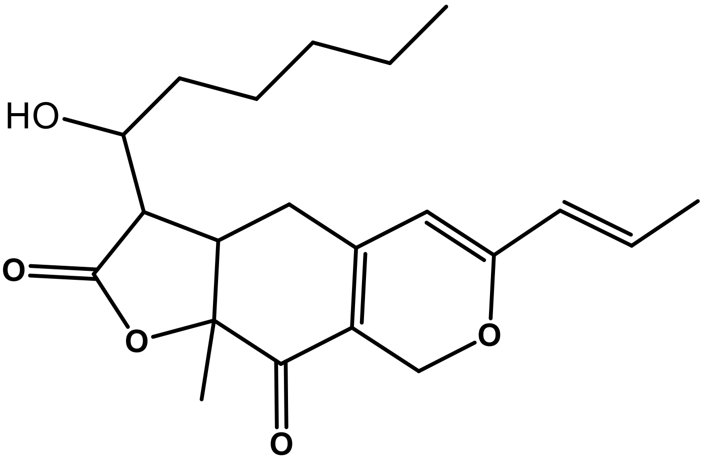

Figure S4. The molecular structure of monascuspiloin ($C_{21}H_{28}O_5$).
